# Solving the Arizona search problem by imputation

**DOI:** 10.1016/j.isci.2024.108831

**Published:** 2024-01-12

**Authors:** Egor Lappo, Noah A. Rosenberg

**Affiliations:** 1 Department of Biology, Stanford University, Stanford, CA, USA

**Keywords:** Molecular genetics, Bioinformatics

## Abstract

An “Arizona search” is an evaluation of the numbers of pairs of profiles in a forensic-genetic database that possess partial or complete genotypic matches; such a search assists in establishing the extent to which a set of loci provides unique identifications. In forensic genetics, however, the potential for performing Arizona searches is constrained by the limited availability of actual forensic profiles for research purposes. Here, we use genotype imputation to circumvent this problem. From a database of genomes, we impute genotypes of forensic short-tandem-repeat (STR) loci from neighboring single-nucleotide polymorphisms (SNPs), searching for partial STR matches using the imputed profiles. We compare the distributions of the numbers of partial matches in imputed and actual profiles, finding close agreement. Despite limited potential for performing Arizona searches with actual forensic STR profiles, the questions that such searches seek to answer can be posed with imputation-based Arizona searches in increasingly large SNP databases.

## Highlights

• An “Arizona search” searches a forensic STR database for pairs of matching profiles
• Imputed STR genotypes recapitulate counts of matching loci seen with known genotypes
• Non-forensic SNP datasets can be used to perform Arizona searches by imputation

## Graphical abstract

**Figure:**
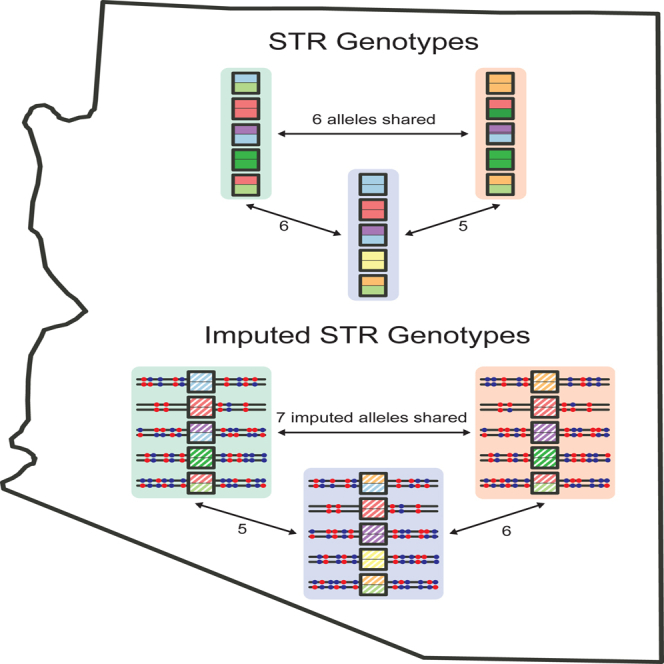

## Introduction

In a common setting in forensic genetics, the genotype of a sample of biological material from an unknown individual is queried against a database of genotypic profiles of known individuals.^1^^,^^2^ The procedure relies on a standardized set of genetic markers typed both in the profiles in the database and in the sample whose identity is sought. A full genotypic match to a database profile can recover the identity of the source of the sample; a partial genotypic match can be informative as well, suggesting that the unknown individual is a relative of the contributor of the partially matching profile.^3^^,^^4^

For the procedure to produce accurate identifications, genotypic profiles across the standardized set of genetic markers must be sufficiently variable that with high probability, a match of a full genotypic profile uniquely identifies an individual across the human species, up to monozygous sibships.^5^ At the same time, it is desirable for the system to possess the fewest loci necessary for establishing uniqueness. The use of a small number of loci minimizes the intrusion of marker systems on genetic privacy, so that profiles contain as little information as possible about individual genotypes and phenotypes; the use of a small number of loci also minimizes the genotyping cost in systems that process many profiles.

What is the minimal size required for a set of loci to achieve the goal that profiles based on that set are unique? As it is impractical to perform the required empirical evaluation—to obtain the genotypes of all possible individuals for a large set of loci, and to choose the optimal subset by analysis of the resulting ginormous dataset—the determination must rely in part on a mathematical model of the level of individual identifiability contained in proposed sets of loci. Indeed, widely used marker sets have been designed using model-based calculations that rely on allele frequencies in small datasets.^6^^,^^7^ In the United States, the set of loci in current use—the “Codis loci,” abbreviated from the “Combined DNA Index System”—has contained 13 highly variable short-tandem-repeat (STR) loci that were first chosen in the 1990s^8^ and that were later augmented with 7 additional loci in 2017.^9^

As profiles on the initial Codis marker set began accumulating in the 1990s, empirical evaluation of the uniqueness of profiles in forensic databases became possible to perform in principle. In such an evaluation, all profiles are compared with all other profiles. The number of pairs of diploid profiles that match at *k* alleles is tabulated, for each value of *k* from 1 to twice the number of loci in the marker set.

Such a pairwise analysis of all profiles in a database has come to be known as an “Arizona search,” after one such evaluation—in which a team working with the forensic profile database for the state of Arizona conducted a search of pairs of profiles in the database.^10^ The analysis identified partial matches at a level that was unexpectedly high—high enough to raise the concern among some that the 13-locus set then in use might not produce a sufficiently high level of uniqueness for individual profiles.^11^^,^^12^

The “Arizona search” incident has had a number of lasting consequences. First, it contributed to the clarification of protocols for forensic databases.^12^^,^^13^ As the purpose of the databases is their operational use for testing query profiles against database profiles, implementation protocols have been clarified so that calculations such as Arizona searches that do not fall into the operational purview generally would not be performed by forensic employees with access to actual profiles.^12^ In the United States, discussions of the possibility for other scientists to access such forensic profiles for research purposes^12^^,^^14^^,^^15^—for example, to conduct “Arizona searches” themselves—have not resulted in such access.

A second consequence was a further understanding of the conceptual meaning of the level of pairwise matching in a forensic query database. The central application of such a database is to assess if some database profile has a match to a profile at hand. The probability that a match exists between two profiles in a database solves a fundamentally different problem—analogous to the probability that two people in a group have a shared birthday rather than the probability that someone in the group has a shared birthday with person *X*.^11^^,^^12^^,^^16^^,^^17^ Nevertheless, the pairwise match probability is informative about the conceptual uniqueness of matches and the fit of probability models to forensic databases.^11^^,^^18^^,^^19^

Finally, recognizing the utility of Arizona searches in understanding the properties of forensic databases, a third consequence is that several studies have sought to provide substitute calculations that mimic a pairwise database search in the absence of access to actual databases. In the model-based Arizona search of Mueller,^18^ independence of a set of forensic loci is assumed. Profiles are generated from allele frequency parameters under independence, producing hypothetical databases. The fraction of profile pairs with complete or partial matches is then obtained. Studies such as that of Mueller^18^ have generally found that models provide a reasonable description of the number of partial matches in databases.

A limitation on such studies is that they use model-based profiles rather than actual profiles. Some studies with sets of actual profiles have been performed,^19^^,^^20^ comparing model-based predictions of the number of pairwise database matches to empirical assessments. Although these studies have tens of thousands of individuals, their numbers of profiles remain small compared to the millions of profiles now present in actual forensic databases. Hence, the potential for understanding pairwise database matches in practical settings continues to rely on mathematical models together with evaluations of the level of empirical matching in smaller datasets.

We and others have recently employed techniques for the imputation of the alleles of forensic STR loci from neighboring SNPs,^21^^,^^22^^,^^23^ introducing a new possibility for evaluating pairwise match probabilities in databases. Non-forensic genomic SNP databases are increasing in size, so that the possibility that millions of SNP profiles will be available for pairwise comparison can be envisioned. With a large database of SNP profiles, the alleles of forensic STRs could conceivably be imputed from the SNPs. From probabilistically imputed STR alleles, the probability of database matches could then be obtained.

An imputation-based calculation enables an Arizona search from SNP profiles, where instead of using a model that generates profiles from allele frequencies, as in the work of Mueller,^18^ the model employed is the imputation model for STR allele probabilities on the basis of the neighboring SNPs. Hence, assuming that the potential for performing Arizona searches from actual STR profiles continues to remain limited, use of imputation in increasingly large SNP datasets can increase the database size for Arizona searches.

In this study, we assess the feasibility of performing an Arizona search of forensic STR profiles by imputation in databases of SNP profiles. We consider individuals for which both SNP and STR genotypes are available. We empirically perform the search using the actual STR profiles, tabulating numbers of partial matches. We then repeat the search by the imputation of STR profiles from SNP profiles, assessing the agreement of the number of partial matches in the imputed data with that in the empirical genotypes. The results suggest that increasingly large SNP databases can indeed be used, together with imputation, to perform searches that mimic Arizona searches of unavailable STR databases.

## Results

### Arizona search with imputed genotypes

We begin by using a dataset of phased SNP–STR genotypes derived from the 1000 Genomes project^23^ to simulate a forensic database (see STAR Methods*:* Data and code availability). We randomly split the 2,504 individuals in the dataset into a *reference panel* (60%, 1,502 individuals) for use in the imputation procedure, and a *database* set (40%, 1,002 individuals), in which the Arizona searches are performed. We consider 100 replicate reference–database splits to ensure that results are not affected by artifacts of random splitting.

For individuals in a database set, we have two kinds of genotype data available: the true STR genotypes at 18 Codis loci, and STR genotypes imputed with the BEAGLE program^24^^,^^25^ using neighboring SNP genotypes and the reference panel (STAR Methods*:* Imputation with BEAGLE). We refer to the imputed genotypes as “BEAGLE-called” genotypes.

For the true genotypes, we calculate the numbers of matching alleles, loci matching at both alleles (“fully matching”), and loci matching at exactly one allele (“partially matching”) for each of $\left(\begin{array}{c} 1002\\ 2 \end{array}\right)=501,501$ possible pairs of individuals.

**Figure 1:**
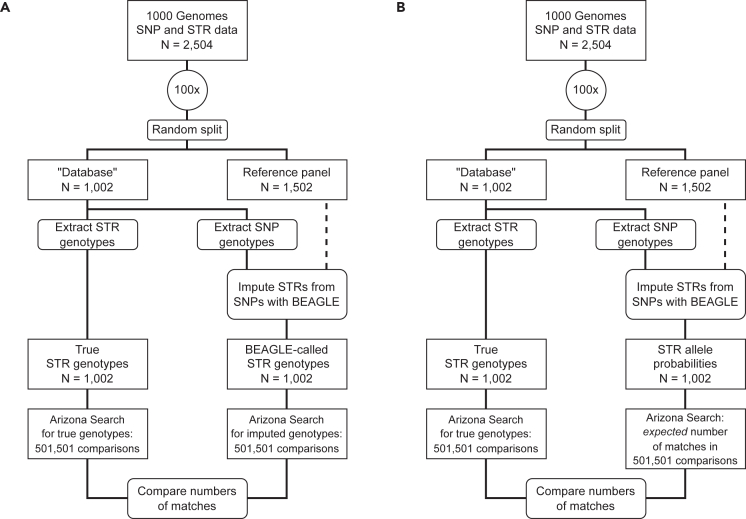
The experimental design Rectangular boxes represent data, rounded boxes represent actions, and circles mean that the actions below are repeated multiple times. (A) Scheme 1: Arizona search using BEAGLE-called genotypes. (B) Scheme 2: Arizona search using STR allele probabilities inferred by BEAGLE for each individual in the database. The 100 replicate splits are the same in Schemes 1 and 2.

We then repeat this calculation for BEAGLE-called genotypes and compare the values obtained with those for true genotypes. We refer to this approach as Scheme 1 (Figure 1A).

### Arizona search with imputed allele probabilities

The BEAGLE-called genotypes do not capture all the information that is produced by the imputation procedure. The imputation algorithm also estimates allele probabilities for each locus on each chromosome for every sample, representing the uncertainty in the imputation. The BEAGLE-called genotypes are then assigned to be alleles with the highest probability.

In a second experiment, working with the same 100 random splits of reference and database samples, we used the estimated allele probabilities directly to compute *expected* numbers of allele and locus matches for each pair of individuals in the database, as described in STAR Methods*:* Expected number of matches. Expected numbers of matches represent the similarity between a pair of individuals across all possible genotype combinations, weighted according to the imputed allele probabilities. We refer to this approach as Scheme 2 (Figure 1B).

### Distributions of numbers of matches

**Figure 2:**
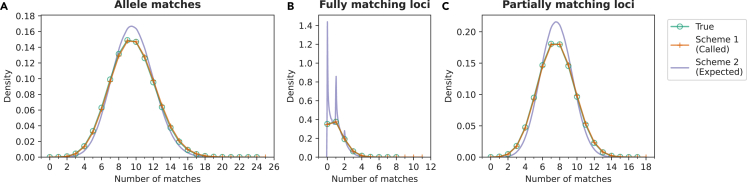
Distributions of the numbers of matching alleles, fully matching loci, and partially matching loci in Arizona searches in simulated forensic databases Normalized histograms are plotted for discrete match counts using true STR genotypes (green) and imputed STR genotypes (Scheme 1, orange). Kernel density estimates are plotted for expected matches (Scheme 2, purple). All 100 replicate splits are combined to produce a single distribution. (A) Number of matching alleles between two individuals. (B) Number of fully matching loci between two individuals. (C) Number of partially matching loci between two individuals.

We perform Arizona searches using true STR genotype data and imputed STR genotype data obtained using Schemes 1 and 2. Figure 2 shows match distributions over $\left(\begin{array}{c} 1002\\ 2 \end{array}\right)$ possible comparisons in the database, averaged over all 100 replicates. The distributions are summarized in Table 1.

**Table 1:** Summaries of distributions of the numbers of matching alleles, fully matching loci, and partially matching loci in Arizona searches in simulated forensic databases

Summary statistic	Variable	True data	Scheme 1	Scheme 2
Median	Matching alleles	10	10	9.602
	Fully matching loci	1	1	0.962
	Partially matching loci	8	8	7.597
Maximum	Matching alleles	24	25	22.906
	Fully matching loci	8	11	8.268
	Partially matching loci	17	18	17.062

Medians and maximal observed values are computed after pooling results on 100 replicate splits of the starting dataset into reference and database samples.

In an Arizona search with the true data, the median number of matching alleles is 10, and the maximal value observed across the replicates is 24. The theoretical maximum is 36, corresponding to a comparison of identical samples. For the counts of fully matching loci, the median of the true distribution is 1, and largest observed value is 8 compared to a theoretical maximum of 18. Finally, for partially matching loci, the median is 8, and the observed maximum is 17.

Both ways of using imputed data produce distributions of matches close to the true data. Arizona search using BEAGLE-called genotypes (Scheme 1) recovers the correct medians (Table 1). Visually, the distributions of the numbers of allele matches, fully matching loci, and partially matching loci are close to the true ones. The range of values is larger with imputed data: most noticeably, the maximal numbers for counts of fully matching loci are 8 and 11 for true and imputed genotypes, respectively.

Using the expected numbers of matches computed from imputed allele probabilities (Scheme 2) yields a distribution of the numbers of allele matches that is more concentrated than the true discrete distributions (Figure 2A). The medians are close to true values, as are the observed maxima (Table 1).

### Match error due to imputation

The Arizona searches using imputed data recover the distributions of allele and locus matches across pairs of individuals; we now evaluate the procedure at the level of specific pairs of individuals.

**Figure 3:**
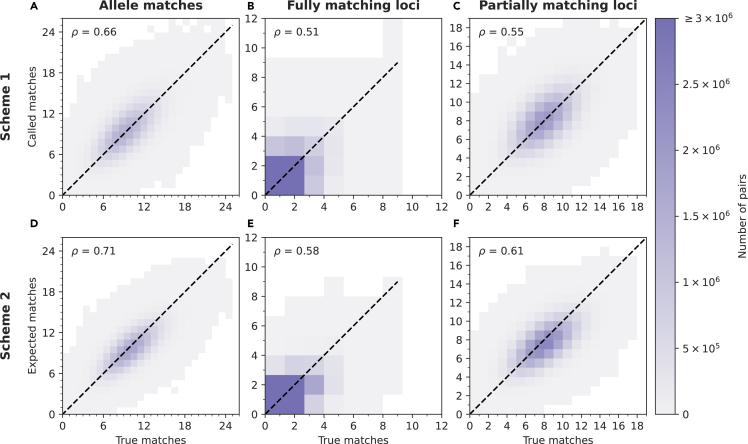
Comparison of numbers of matches with imputed and true data for all pairs of individuals in the database In each panel, the *x*-axis is the number of matching alleles or loci with true STR genotype data, and the *y* axis shows the corresponding number with imputed data. The Spearman correlation coefficient ρ is shown for each panel. The panels show matches in all 100 replicates combined into a single distribution. In the figure, for integers (x,y), the unit square centered at $\left(x+\frac{1}{2},y+\frac{1}{2}\right)$ depicts values in [x,x+1)×[y,y+1). (A) Scheme 1, allele matches. (B) Scheme 1, fully matching loci. (C) Scheme 1, partially matching loci. (D) Scheme 2, allele matches. (E) Scheme 2, fully matching loci. (F) Scheme 2, partially matching loci.

Figure 3 compares the numbers of matches for true and imputed data for each pair of individuals. The numbers computed using Scheme 1 are reasonably correlated with the true values (Spearman correlations of 0.66, 0.51, and 0.55 for allele matches, fully matching loci, and partially matching loci, respectively). In each category of matches, for more than 50% of pairs, the absolute difference between the number of matches in Scheme 1 and the true number is no more than 1. In 90% of pairs, Scheme 1 differs from true values by 3 or less (Table 2).

**Table 2:** Absolute difference between the number of matches in Schemes 1 and 2 and the true values

Summary statistic	Variable	Scheme 1	Scheme 2
Median absolute error	Matching alleles	1	1.237
	Fully matching loci	1	0.491
	Partially matching loci	1	1.149
90th percentile of the absolute error	Matching alleles	3	3.070
	Fully matching loci	2	1.361
	Partially matching loci	3	2.834

The differences are computed after merging results on 100 independent replicate splits of the starting dataset into reference and database samples.

Scheme 2 increases the agreement of the algorithm with the true values. Correlations of true and expected numbers of matches are higher (0.71, 0.58, 0.61 for allele matches, fully matching loci, and partially matching loci). Median absolute error is also near one allele or locus (Table 2).

To further characterize the differences between true numbers of matches and those computed with imputed data, we use the Hodges-Lehmann estimator of the difference of means for paired samples.^26^ Let $T_{i}$ be the true number of matches (for any of the three match categories) and let $I_{i}$ be the number of matches with imputed data (with either Scheme 1 or 2), for i=1,2,…,501501. Let $E_{i}=I_{i}-T_{i}$. Rearrange the $E_{i}$ in non-decreasing order, $E_{1}\leq E_{2}\leq \cdots \leq E_{501501}$. Our estimate of the difference between numbers of imputed and true matches is the median of averages of all pairs in the set $\left\{E_{i}\right\}$:

(Equation 1) $$\hat{\theta }=\text{median}\left\{\frac{E_{i}+E_{j}}{2}\;|\;i<j\right\}\text{.}$$

The value of $\hat{\theta }$ is an estimator that is well suited to our problem, as it does not introduce any assumptions on the distributions of the numbers of matches and it is robust to outliers.

**Figure 4:**
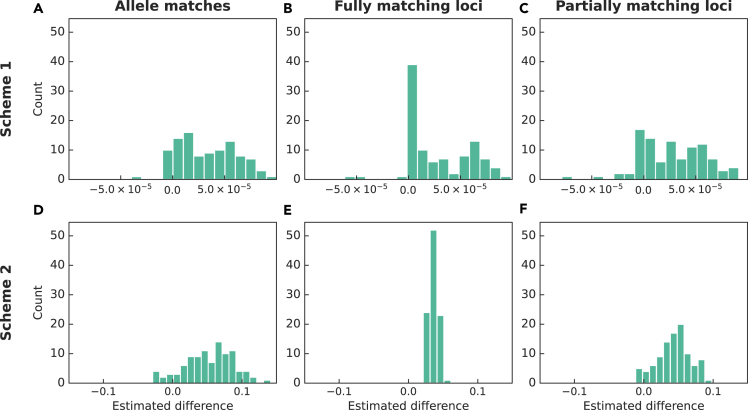
Differences between the numbers of matches with imputed and true data, computed using the Hodges-Lehmann estimator The histograms show distributions across the 100 replicate splits. (A) Scheme 1, allele matches. (B) Scheme 1, fully matching loci. (C) Scheme 1, partially matching loci. (D) Scheme 2, allele matches. (E) Scheme 2, fully matching loci. (F) Scheme 2, partially matching loci.

The Hodges-Lehmann estimates, shown in Figure 4 as distributions over 100 replicate splits, lie in [−0.15,0.15]. Hence, on average, using called genotypes (Scheme 1) or expected matches (Scheme 2) computed from SNP data biases the Arizona search results by less than 0.15 of a match.

## Discussion

We have analyzed the possibility of performing Arizona searches of STR databases using SNP genotype data and imputation. Using 18 of the 20 Codis STR loci and neighboring SNPs, we have described Arizona searches by imputation that use either most likely STR genotype assignments (Scheme 1) or STR allele probabilities (Scheme 2) obtained by imputation using surrounding SNPs (Figure 1).

Both schemes recover the true distributions of the numbers of matching alleles and loci (Figure 2), and the medians of three classes of matches closely agree with the true values. For the maximal number of matches, Scheme 2 provides values close to those of the true data; Scheme 1 sometimes yields pairs with higher numbers of matches (Table 1). That Scheme 1 would not perform as well on this metric is sensible: although the calculation using imputed allele probabilities reasonably captures the uncertainty in the imputation algorithm, Scheme 1 is systematically biased toward selecting more probable (and more frequent) alleles for each individual, increasing the probability of observing pairs with high numbers of matches.

When specific pairs of individuals are considered, the median absolute error in the number of matches computed by imputation is near 1 (Table 2). Correlations between numbers of imputed and true matches are reasonably high (Figure 3), though error can be nontrivial for specific pairs. As in other imputation studies,^27^^,^^28^ it is likely that some of this error can be eliminated with larger reference panels.

As forensic genetics has been increasingly examining new SNP sets that could eventually augment or even replace existing STR systems,^29^^,^^30^ it is possible that the Arizona search question of understanding the distribution of pairwise agreement among profiles will become relevant for new potential marker sets. Although we have focused here on imputing STRs from SNPs, imputation of the relevant SNPs in proposed marker sets from neighboring SNPs could proceed similarly, and indeed would be more similar than our present SNP–STR analysis to typical biomedical imputations of SNPs from other SNPs.

Imputation has appeared in a variety of problems in forensic genetics;^21^^,^^22^^,^^23^^,^^31^^,^^32^^,^^33^^,^^34^ its use for the Arizona search problem is one of an increasing number of scenarios in which loci external to forensic systems can assist in understanding forensic genetic matching. Imputation has enabled the matching of genetic records between profiles of SNP loci and profiles of STR loci, potentially linking SNP and STR databases in principle.^21^^,^^22^^,^^33^ It can also help in testing STR loci for phenotypic associations while attempting to understand the phenotypes that might be associated with particular forensic profiles.^31^^,^^34^

### Limitations of the study

Our somewhat simplistic analysis in the 1000 Genomes—a dataset with relatively few individuals compared to that in which the largest reported Arizona search has been performed^19^—provides a demonstration that the imputation-based Arizona search approach is feasible. However, we note a number of limitations. First, the 1000 Genomes SNP–STR haplotype panel we used was itself obtained using imputation based on an external family-based reference dataset.^23^ While the accuracy of this procedure was found to be high,^23^ imputation errors could still be present in the data. It is important to be cautious in interpreting our computations for any particular pair of individuals, and it will be useful to perform similar analyses in datasets containing SNP and STR genotypes obtained directly. We note also that we have not taken into account population structure among profiles in the database of profiles; a future direction is to examine imputation in the context of approaches to Arizona searches incorporating the Balding–Nichols model that takes population structure into account.^19^ The possibility that the database contains siblings, parents and offspring, or other close relatives could also be considered.

Finally, we note that in our analysis of the 1000 Genomes data, we are relying on an assumption that a forensic database accurately represents the profiles of its sampled individuals. Genotyping errors, recording errors, sample mislabelings, and sample duplications can alter the relationship between the set of individuals for whose profiles an Arizona search is of interest and the actual profiles employed in such a search. Such factors will be important to consider in interpreting any imputation-based Arizona searches performed beyond the controlled scenario of a simulation.

## STAR★Methods

### Key resources table

**Table:** 

REAGENT or RESOURCE	SOURCE	IDENTIFIER
**Software and algorithms**		

BEAGLE v5.4	Browning, Zhou, and Browning^24^	http://faculty.washington.edu/browning/beagle/beagle.html
Custom computer code	This paper	https://github.com/EgorLappo/arizona-searches-by-imputation

**Other**		

Genotype data	Kim and Rosenberg,^33^ Saini et al.^23^	https://github.com/jk2236/RM_WGS

### Resource availability

#### Lead contact

The lead contact for requests for resources is Noah A. Rosenberg (noahr@stanford.edu).

#### Materials availability

None.

#### Data and code availability

• The dataset is a panel of phased SNP–STR haplotypes from Saini et al.,^23^ derived from the 1000 Genomes phase 3^35^ and used previously by Kim & Rosenberg.^33^ It contains STR genotypes for 2,504 individuals at 18 of 20 Codis loci (CSF1PO, D10S1248, D12S391, D13S317, D18S51, D19S433, D1S1656, D22S1045, D2S1338, D2S441, D3S1358, D5S818, D7S820, D8S1179, FGA, TH01, TPOX, vWA). As in Kim & Rosenberg,^33^ we include SNP haplotypes only in 1Mb windows centered on Codis STR loci. The 1000 Genomes data used in the study are available from Saini at al.,^23^ as processed by Kim & Rosenberg^33^; processed data are available from github.com/jk2236/RM_WGS. Processed data are publicly accessible in the supplementary files.
• Code to replicate the analysis and generate figures is available in the supplementary files.
• Any additional information required to reanalyze the data reported in this paper is available from the lead contact upon request.

### Method details

#### Imputation with BEAGLE

We ran the imputation with BEAGLE 5.4^24^ using the human reference genome GRCh37 genetic map and all numerical parameters set to default values, as in Kim & Rosenberg.^33^ The parameter values we used are^36^: impute=true, ap=true, gp=true, imp-states=1600, imp-segment=6.0, imp-step=0.1, imp-nsteps=6.0, cluster= 0.005, ne=100000, window=40.0, overlap=2.0, seed=-99999.

#### Expected number of matches

To compute the number of matches expected between two imputed profiles in Scheme 2, we derive exact expressions for the distributions of the numbers of fully and partially matching loci for a pair of individuals given allele probabilities at each locus.

Suppose we have run the BEAGLE imputation algorithm for an STR locus l of an individual *A*. The output contains two allele probability vectors $A_{1}$ and $A_{2}$ for the two haplotypes of *A*, each of length $N_{l}$—the number of distinct alleles of l observed in the reference panel. The probability of observing a given ordered genotype i∣j is the product $A_{1,i}A_{2,j}$. We can use these probabilities to compute distributions of the numbers of fully and partially matching loci between any two individuals. We first consider a single locus l, and we then extend to any number of loci by dynamic programming.

Suppose we have two individuals *A* and *B* and four probability vectors $A_{1}$, $A_{2}$, $B_{1}$, and $B_{2}$ for alleles at a locus l. Given the genotype probabilities for *A* and *B*, what are the probabilities that *A* and *B* match fully (at both copies) and partially (exactly one allele is the same for *A* and *B*)? Let $P_{1,0}^{l}$ be the probability of a full match, $P_{0,1}^{l}$ of a partial match, and $P_{0,0}^{l}$ of a non-match.

To find an expression for $P_{1,0}^{l}$, we work case-by-case for each possible genotype of individual *A*. We consider cases of i≠j and i=j separately. First, if i=j, then genotypes of *A* and *B* must both be i∣i, which happens with probability

(Equation 2) $$\sum_{i=1}^{N_{l}}A_{1,i}A_{2,i}B_{1,i}B_{2,i}\text{.}$$

If i≠j, then there are two possible cases: the match is A:i∣j, B:i∣j, or the match is A:i∣j, B:j∣i. Together, the two cases have probability

(Equation 3) $$\sum_{i=1}^{N_{l}}\sum_{j=1,j\neq i}^{N_{l}}A_{1,i}A_{2,j}\left(B_{1,i}B_{2,j}+B_{1,j}B_{2,i}\right)\text{.}$$

The sum of Equations 2 and 3 is the probability of the full match between the two individuals:

(Equation 4) $$P_{1,0}^{l}=\sum_{i=1}^{N_{l}}\sum_{j=1,j\neq i}^{N_{l}}A_{1,i}A_{2,j}\left(B_{1,i}B_{2,j}+B_{1,j}B_{2,i}\right)+\sum_{i=1}^{N_{l}}A_{1,i}A_{2,i}B_{1,i}B_{2,i}\text{.}$$

To compute a probability of a partial match, we again consider i=j, so that the genotype of *A* is i∣i. A partial match happens if *B* has genotype i∣k or k∣i with k≠i. The resulting probability is

$$\sum_{i=1}^{N_{l}}A_{1,i}A_{2,i}\left[B_{1,i}\left(1-B_{2,i}\right)+\left(1-B_{1,i}\right)B_{2,i}\right]\text{.}$$

If i≠j, then a partial match corresponds to the following cases: A:i∣j, B:i∣k; A:i∣j, B:j∣k; A:i∣j, B:k∣i; and A:i∣j, B:k∣j, all with k≠i, k≠j. These cases have probability

$$\sum_{i=1}^{N_{l}}\sum_{j=1,j\neq i}^{N_{l}}A_{1,i}A_{2,j}\left[B_{1,i}\left(1-B_{2,j}\right)+B_{1,j}\left(1-B_{2,i}\right)+\left(1-B_{1,i}-B_{1,j}\right)\left(B_{2,i}+B_{2,j}\right)\right]\text{.}$$

Together, we get

(Equation 5) $$P_{0,1}^{l}=\sum_{i=1}^{N_{l}}\sum_{j=1,j\neq i}^{N_{l}}A_{1,i}A_{2,j}\left[B_{1,i}\left(1-B_{2,j}\right)+B_{1,j}\left(1-B_{2,i}\right)+\left(1-B_{1,i}-B_{1,j}\right)\left(B_{2,i}+B_{2,j}\right)\right]+\sum_{i=1}^{N_{l}}A_{1,i}A_{2,i}\left[B_{1,i}\left(1-B_{2,i}\right)+\left(1-B_{1,i}\right)B_{2,i}\right]\text{.}$$

Finally, we use a similar approach for calculating the probability of a non-match. Here, the individual genotypes are A:i∣j, B:k∣l with k≠i, k≠j, l≠i, l≠j, giving the following expression:

(Equation 6) $$P_{0,0}^{l}=\sum_{i=1}^{N_{l}}\sum_{j=1,j\neq i}^{N_{l}}A_{1,i}A_{2,j}\left(1-B_{1,i}-B_{1,j}\right)\left(1-B_{2,i}-B_{2,j}\right)+\sum_{i=1}^{N_{l}}A_{1,i}A_{2,i}\left(1-B_{1,i}\right)\left(1-B_{2,i}\right)\text{.}$$

Now suppose that we have imputed *L* STR loci for individuals *A* and *B*. Following our previous analyses that have assumed that imputation proceeds independently at different STR loci,^21^^,^^22^^,^^33^ suppose also that the match probabilities are independent between loci (i.e. linkage equilibrium). To convert one-locus results into a many-locus model, we use the recursive equations of Tvedebrink et al.^20^ Let $\pi_{m,p}^{l}$ be the probability of observing *m* full matches and *p* partial matches when considering the first l of *L* loci; if m+p>l, then $\pi_{m,p}^{l}=0$. The initial conditions are $\pi_{1,0}^{1}=P_{1,0}^{1}$, $\pi_{0,1}^{1}=P_{0,1}^{1}$, and $\pi_{0,0}^{1}=P_{0,0}^{1}$. Other values are calculated recursively as

(Equation 7) $$\pi_{m,p}^{l+1}=\left\{ \begin{array}{ll} P_{0,0}^{l+1}\pi_{m,p}^{l}+P_{0,1}^{l+1}\pi_{m,p-1}^{l}+P_{1,0}^{l+1}\pi_{m-1,p}^{l} & \text{if}\;m>0,p>0,\\ P_{0,0}^{l+1}\pi_{0,p}^{l}+P_{0,1}^{l+1}\pi_{0,p-1}^{l} & \text{if}\;m=0,p>0,\\ P_{0,0}^{l+1}\pi_{m,0}^{l}+P_{1,0}^{l+1}\pi_{m-1,0}^{l} & \text{if}\;m>0,p=0,\\ P_{0,0}^{l+1}\pi_{0,0}^{l} & \text{if}\;m=0,p=0. \end{array}\right.$$

The values $\pi_{m,p}^{L}$ characterize the discrete bivariate distribution of fully and partially matching loci between individuals *A* and *B*, conditional on the BEAGLE-estimated probabilities of individual alleles. We obtain expected numbers of fully and partially matching loci as $\sum_{m=0}^{L}\sum_{p=0}^{L-m}m\pi_{m,p}^{L}$ and $\sum_{m=0}^{L}\sum_{p=0}^{L-m}p\pi_{m,p}^{L}$, respectively. The expected number of matching alleles is $\sum_{m=0}^{L}\sum_{p=0}^{L-m}\left(2m+p\right)\pi_{m,p}^{L}$.

## Supplemental information

Data S1. Processed data and code to replicate the analysis
