# Supplementary figures and images for: Solving the Arizona search problem by imputation
Source: iScience. 2024 Jan 12;27(2):108831. doi: 10.1016/j.isci.2024.108831 (PMC10845060; doi:10.1016/j.isci.2024.108831)

Scheme 1

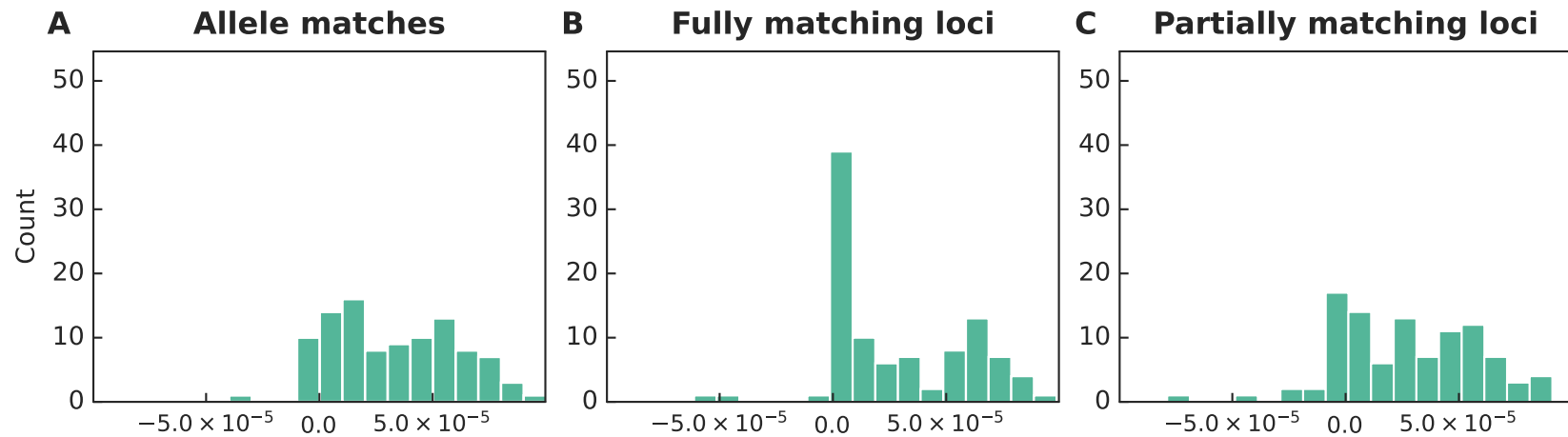

Scheme 2

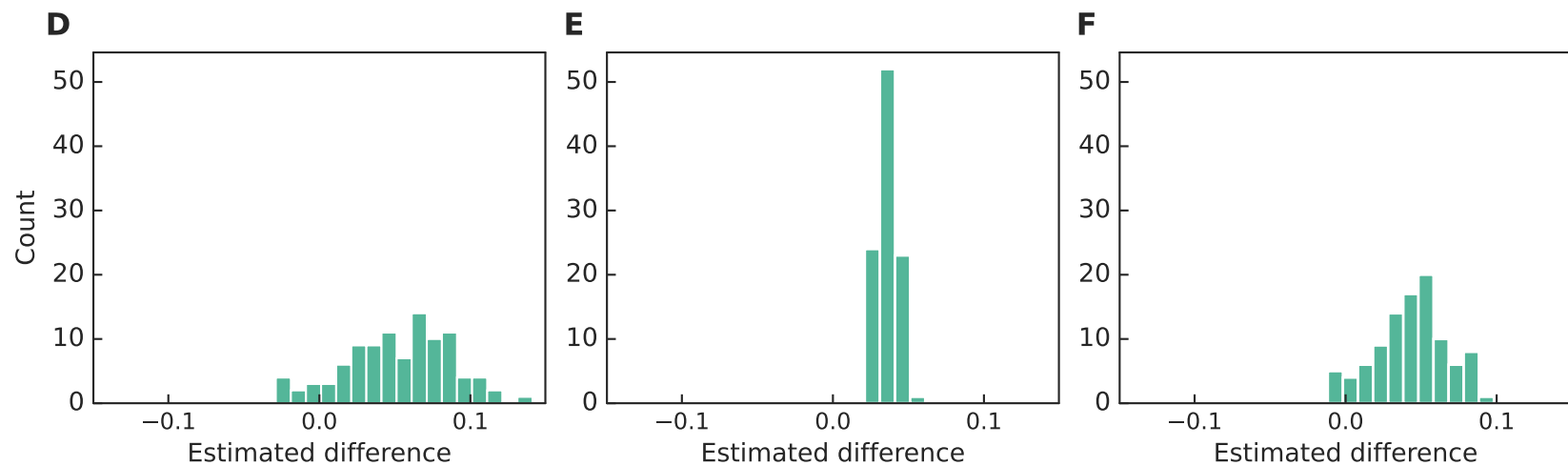

Supplement: Data S1. Processed data and code to replicate the analysis [file mmc1.zip › arizona-searches-by-imputation/figures/wilcoxon_effect_sizes.pdf]

**Scheme 1**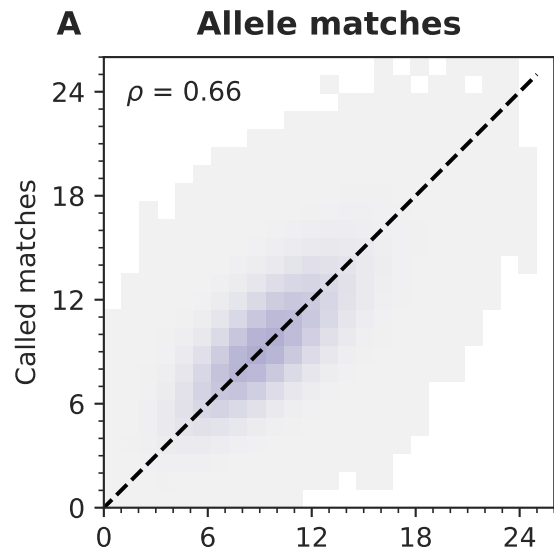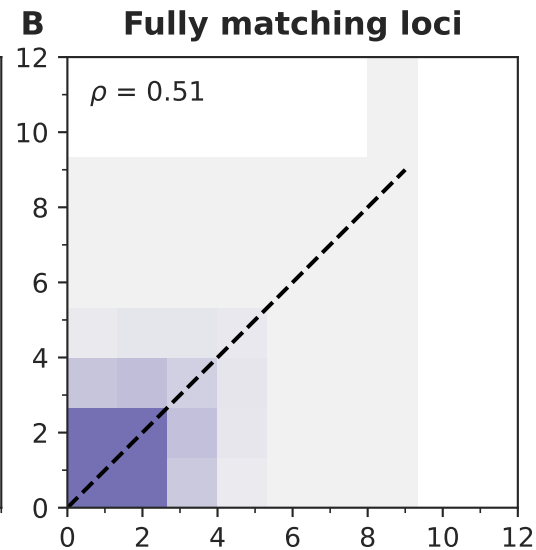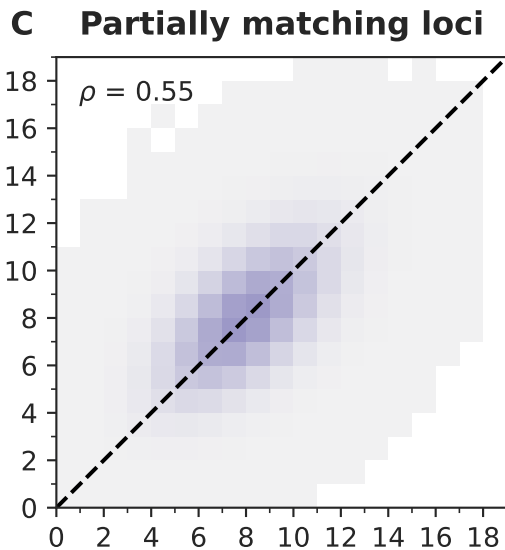**Scheme 2**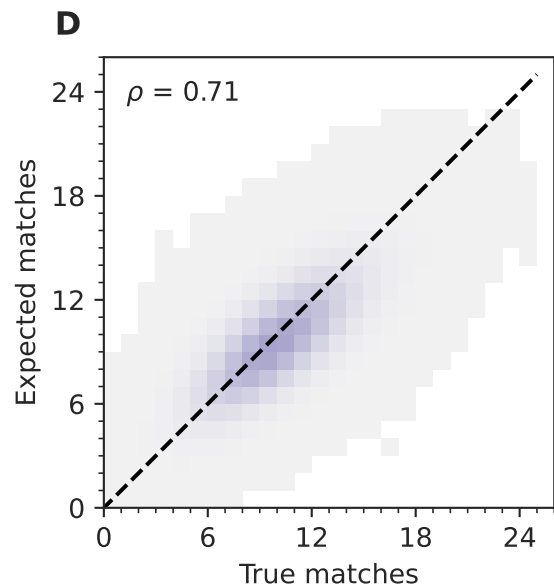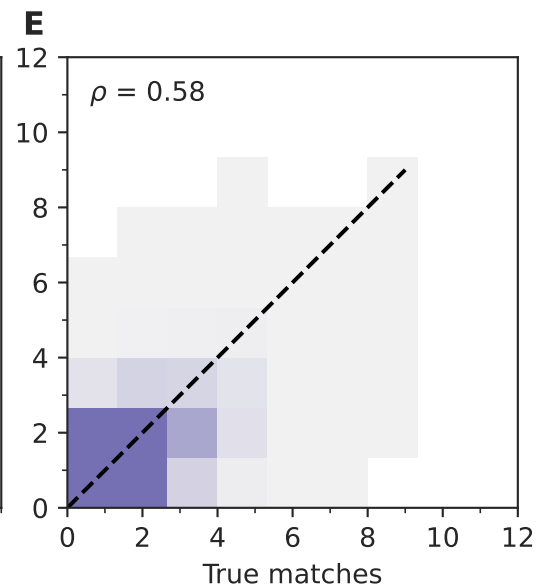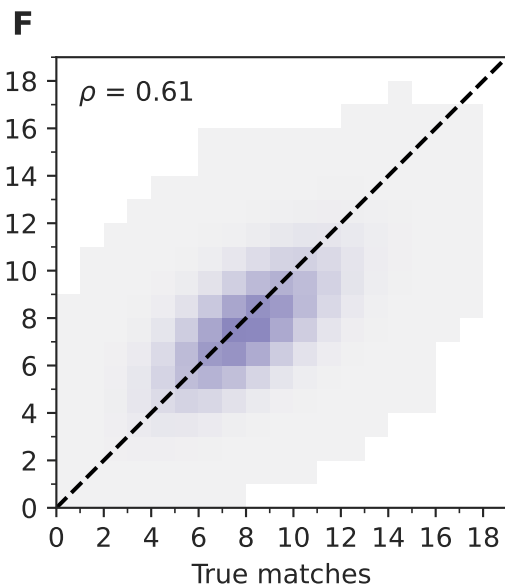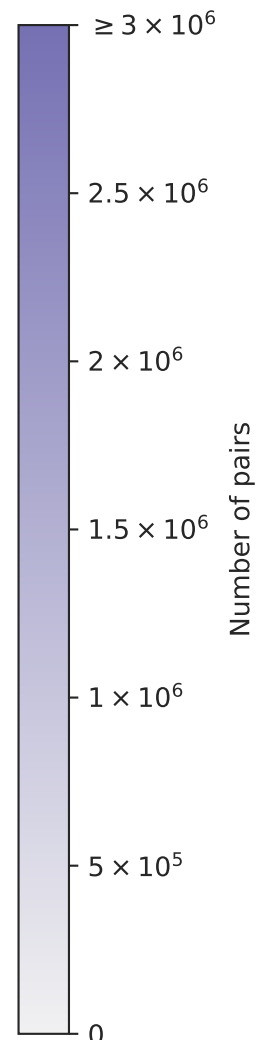

Supplement: Data S1. Processed data and code to replicate the analysis [file mmc1.zip › arizona-searches-by-imputation/figures/matches_correlation.pdf]

**A****Allele matches**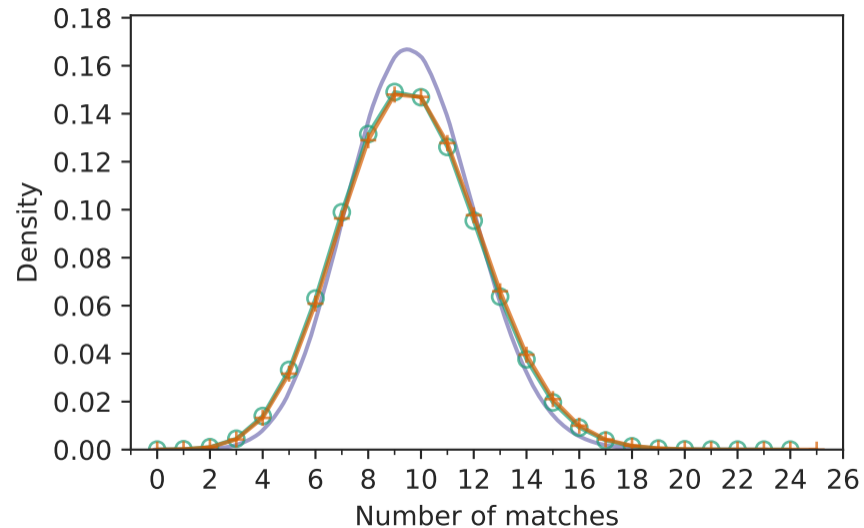**B****Fully matching loci**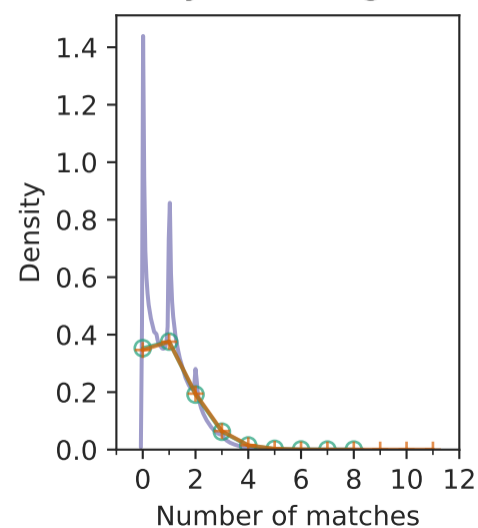**C****Partially matching loci**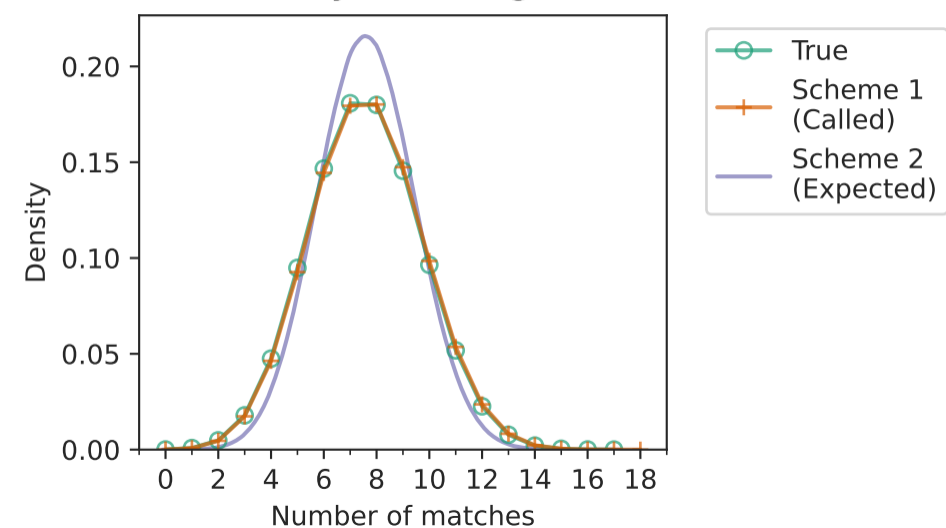

Supplement: Data S1. Processed data and code to replicate the analysis [file mmc1.zip › arizona-searches-by-imputation/figures/match_densities.pdf]
